# The Simple Lamb Wave Analysis to Characterize Concrete Wide Beams by the Practical MASW Test

**DOI:** 10.3390/ma9060437

**Published:** 2016-06-02

**Authors:** Young Hak Lee, Taekeun Oh

**Affiliations:** 1 Department of Architecture Engineering, Kyung Hee University, 1732 Deogyeong-daero, Giheung-gu, Yongin 17104, Korea; leeyh@khu.ac.kr; 2 Department of Safety Engineering, Incheon National University, 12-1 Songdo-dong, Yeonsu-Gu, Incheon 22012, Korea

**Keywords:** Lamb wave, MASW, surface wave, Rayleigh wave, P-wave, condition assessment, concrete, thickness estimation

## Abstract

In recent years, the Lamb wave analysis by the multi-channel analysis of surface waves (MASW) for concrete structures has been an effective nondestructive evaluation, such as the condition assessment and dimension identification by the elastic wave velocities and their reflections from boundaries. This study proposes an effective Lamb wave analysis by the practical application of MASW to concrete wide beams in an easy and simple manner in order to identify the dimension and elastic wave velocity (R-wave) for the condition assessment (e.g., the estimation of elastic properties). This is done by identifying the zero-order antisymmetric (A0) and first-order symmetric (S1) modes among multimodal Lamb waves. The MASW data were collected on eight concrete wide beams and compared to the actual depth and to the pressure (P-) wave velocities collected for the same specimen. Information is extracted from multimodal Lamb wave dispersion curves to obtain the elastic stiffness parameters and the thickness of the concrete structures. Due to the simple and cost-effective procedure associated with the MASW processing technique, the characteristics of several fundamental modes in the experimental Lamb wave dispersion curves could be measured. Available reference data are in good agreement with the parameters that were determined by our analysis scheme.

## 1. Introduction

Concrete has been a popular construction material due to its economic efficiency and good durability. As such, concerns about concrete structures, with regard to their quality assurance (in terms of the strength, elasticity and density) and their condition assessment (in terms of dimension identification and the damage characterization of delamination, cracks and voids), are important to consider [1,2]. Various non-destructive evaluation (NDE) methods, as a solution to these types of issues, have been proposed and developed [3]. Among these, stress wave-based testing techniques have the advantage of allowing one to determine certain fundamental material properties and dimensions, as well as identify flaws, in a simple and effective manner [4]. As an example of a stress wave-based NDE, the impact-echo (IE) method, which uses multiple body wave reflections from the crack or bottom surface, has been widely applied to concrete structures in order to determine dimensions and to characterize damage. In the IE measurement, the tested structure is subjected to a transient impact. The subsequent transient dynamic response is recorded, and the resonance frequency of the structure is determined. The resonance frequency corresponding to the impact-echo mode (or the thickness mode), and the indirectly-measured P-wave velocity can indicate certain material properties, as well as the thickness or existence of flaws [5]. Alternatively, the ultrasonic testing method, which utilizes the velocity and reflections of pressure, shear and Rayleigh waves (P-, S- and R-waves), can provide useful information about the dimensions and structural integrity. Initially, the P-wave was commonly applied to concrete structures, but the application of S- or R-waves has gradually increased because these are less sensitive to confinement and boundary conditions; they also have a larger energy magnitude than P-waves [6].

For plate-like structures, such as concrete slabs and asphalt pavements, the IE and ultrasonic testing methods can be effectively applied, and the combination of these two techniques can give more accurate information. In this regard, many researchers have studied the fusion of various NDE methods. Kee *et al.* [7] proposed combining impact-echo and infrared thermography for damage identification in the deck slabs of bridges. Baggens and Ryden [8] presented the possibility of a combined impact-echo and surface wave measurement to analyze plate-like structures, such as asphalt pavement, and to identify Lamb wave plate parameters, such as Poisson’s ratio, because the asphalt has a broad range of Poisson’s ratio due to the high sensitivity of temperature. They utilized both the Rayleigh wave velocity from A0 Lamb mode by the MASW test and the ratio of S1-ZGV (zero group velocity) Lamb mode frequency to the A2-ZGV one by the impact-echo test for the estimation of Poisson’s ratio. These efforts have contributed greatly to the development of NDE methods for plate-like structures; however, they still have some limitations in practical field applications, including their complicated interpretation, laborious testing and indirect information.

This study focuses on the simple Lamb wave analysis by the practical application of MASW, combining the IE method and the Rayleigh wave velocity measurement, which utilizes simple equipment and a facile analysis process without the separate impact-echo test. The proposed MASW technique yields effective phase dispersion curves not only of the plate-like one, such as the slab and pavement, but also of the semi-plate-like structures, such as the wide-beam, by minimizing the effects of reflections from edge boundaries through the coherence of many data acquisitions associated with the multiple sensors and instrumented impact hammer with a high frequency range in a test setup. Among various guided Lamb modes, the A0 and S1 modes, which correspond to the Rayleigh wave velocity and impact-echo mode, respectively, were easily and clearly identified. In a field test, the A0 and S1 modes extracted from dispersion curves (made from field measurements) can be used to characterize a material or provide quality assurance. Moreover, this information can be used to make calibration values for the elastic properties and thickness, which is useful in conventional ultrasonic testing, especially if the thickness of a structure is not known in advance. Furthermore, the entire testing procedure can be completed in an hour with cost-effective equipment. Both measurements and analysis are made on the same portable computer, and the recorded data can be evaluated directly in the field to obtain the final results. The experimental results present practical guidelines for applying stress wave-based NDE methods to concrete structures.

## 2. Lamb Wave Modes and Dispersion Curves

**Figure 1:**
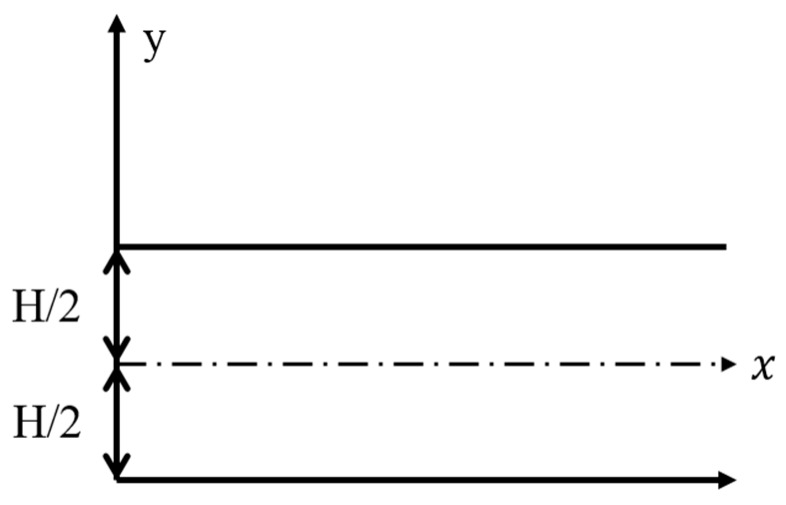
Schematic geometry of a free plate problem with regard to the Lamb wave.

The dispersion curve obtained by the MASW in this study is based on the principles of Lamb wave theory. Lamb waves [9] are a type of guided dispersive waves propagating in elastic and isotropic plate structures with traction-free boundaries. Lamb waves formed by the interference of multiple reflections and the mode conversion of P- and S-waves at the free surfaces of the plate are presented in Figure 1 [10]. Typically, by matching theoretical multimodes of Lamb wave dispersion curves with experimental ones, the shear (V_S_) and Raleigh wave (V_R_) velocities, Poisson’s ratio (v) and the thickness of the tested plate structure can be determined. Lamb waves have been used in various NDT applications, such as material characterization of elastic plates [11,12], defect inspection [13] and thickness measurements of thin films [14].

Lamb waves are a group of wave types including the bending wave, the Rayleigh wave and the quasi-longitudinal wave. Lamb [9] organized the dispersion equation that manages the transitions between these types of waves. Harmonic wave propagation in the x-direction can be represented as combinations of the frequency (*f*) and phase velocity (*C_P_*) that correspond to standing waves in the thickness direction (*y*-direction). These waves must obey the following dispersion Equations [9]:

(1) $$\frac{\tan\;(q\frac{H}{2})}{\tan\;(p\frac{H}{2})}=-\frac{4k^{2}pq}{{\left(q^{2}-k^{2}\right)}^{2}}\text{ for symmetric modes}$$

(2) $$\frac{\tan\;(q\frac{H}{2})}{\tan\;(p\frac{H}{2})}=-\frac{{\left(q^{2}-k^{2}\right)}^{2}}{4k^{2}pq}\text{ for symmetric modes}$$

Here, *p* and *q* are given by:

(3) $$p^{2}={\left(\frac{\omega }{V_{P}}\right)}^{2}-k^{2}$$

(4) $$q^{2}={\left(\frac{\omega }{V_{S}}\right)}^{2}-k^{2}$$

The wavenumber *k* is numerically equal to *ω*/*C_P_*, where *C_P_* is the phase velocity of the Lamb wave mode and *ω* is the circular frequency. The phase velocity is related to the wavelength by *C_P_* = (*ω*/2*π*)/*λ*. V_P_ and V_S_ are the pressure and shear wave velocities, respectively.

**Figure 2:**
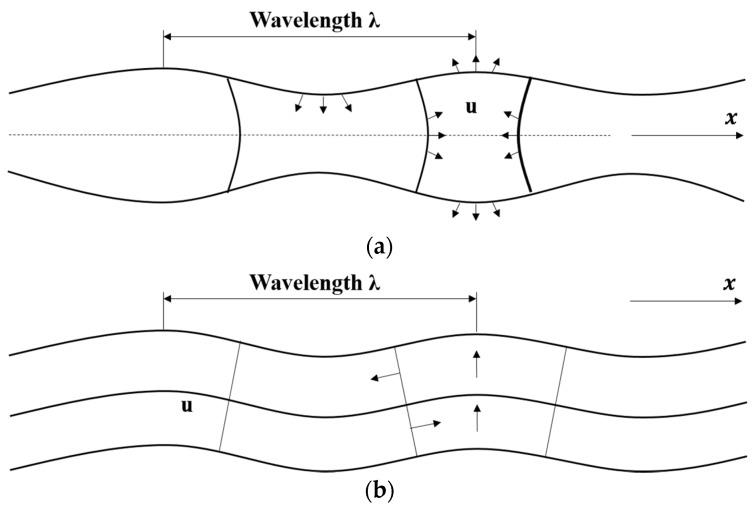
Mode shapes of symmetric (S0) and antisymmetric (A0) fundamental Lamb wave modes. (**a**) Symmetric mode; (**b**) antisymmetric mode.

The dispersion relation is a transcendental function; it is not straightforward to calculate a *k* value at any given frequency. A root-searching technique must be used to find the correct wave numbers at any given frequency [15]. By extracting roots over a wide frequency range, dispersion curves of both symmetric (S) and antisymmetric (A) Lamb waves can be studied. Figure 2 shows the mode shapes of symmetric (S0) and antisymmetric (A0) fundamental Lamb wave modes. Longitudinal displacements of symmetric and antisymmetric wave modes are equal and opposite, respectively, on either side of the median plane. Alternatively, transverse displacements are opposite and equal, respectively [16].

**Figure 3:**
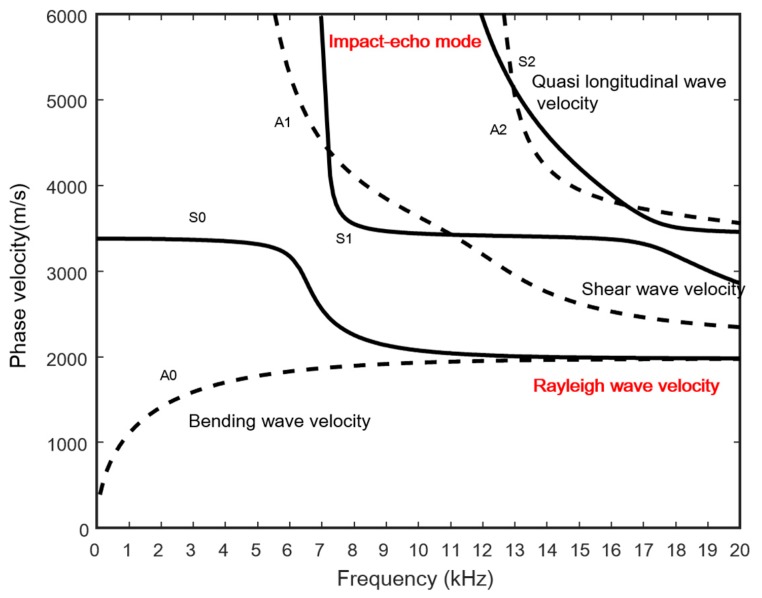
Phase velocity dispersion curves determined by MASW (based on the FE simulation of a concrete slab with a thickness of 0.3 m). A density of 2250 kg/m^3^, an elasticity of 23 GPa and a fixed Poisson’s ratio of 0.167 are assumed for the concrete slab.

Figure 3 presents the dispersion curve of a concrete slab with elastic properties corresponding to normal-strength concrete. At the lowest frequencies (<5 kHz), only the two fundamental modes A0 and S0 exist. In this frequency range, the S0 dispersion curve approaches the quasi-longitudinal wave velocity, which is the P-wave velocity along the plate. The A0 dispersion curve approaches the bending wave velocity of the plate: 300 m/s to 400 m/s at these low frequencies. At higher frequencies, A0 and S0 approach the Rayleigh wave velocity of the plate [17]. It can be shown analytically that Equations (1) and (2) reduce to the Rayleigh wave dispersion equation in a homogeneous half space when the frequency approaches infinity. Typically, in this example, Rayleigh wave motion develops at a range of 10 to 12 kHz; this is where A0 and S0 merge together at the Rayleigh wave velocity. Gibson and Popovics [18] showed that the impact-echo resonance actually corresponds to the S1 mode Lamb wave at the zero group velocity (ZGV) frequency condition. Thus, the S1 -ZGV mode frequency can be used to estimate the plate thickness. In Figure 3, the impact-echo frequency corresponds to 7 kHz in the S1 mode, and the thickness is calculated to be 0.3 m by using the impact-echo equation (Equation (5)). This indicates that the slab thickness H is related to the P-wave velocity V_P_ and the impact-echo frequency *f_IE_*.

(5) $$H=\frac{\beta \;V_{P}}{2f_{IE}}$$

For plate-like structures, *β* is approximately 0.96 [5].

At these high frequencies and small wavelengths, the finite-thickness plate appears as a semi-infinite medium, and wave propagation is limited to the regions closest to the surfaces. Higher modes develop at their respective cut-off frequencies, which are related to the thickness of the plate [19]. At higher frequencies, all higher modes approach the shear wave velocity of the plate. It should also be noted that all symmetric modes have an almost straight portion where the phase velocity is close to the quasi-longitudinal wave velocity.

## 3. Measurement of the R-Wave Velocity and Thickness by MASW

The conventional MASW measurement for the dispersion curve of Lamb waves in the plate is based on N-wave signals collected on the surface along a linear array of sensed points that are equally spaced (with spacing dx) from the wave source [20]. Multiple data are processed as individual signals obtained by each sensor. Data are transformed from the offset time domain to the frequency phase velocity domain using a 2D Fourier transform, which produces a phase velocity that is typically referred to as the dispersion curve [21].

In this study, an effective testing procedure that is associated with the frame equipped with all of the sensors was applied to the MASW measurement. Using this technique, improved dispersion curves could be measured; this is referred to as “multichannel data acquisition with a frame system (MDAFS)”. The multimodal nature of Lamb wave propagation implies that a transient source (e.g., an impact hammer) at the surface will easily generate multiple modes of propagation. Measured time histories along the surface will be composed of a number of superposed modes. Therefore, it is important that different modes of Lamb wave propagation be fulfilled with a practical and cost-effective approach.

**Figure 4:**
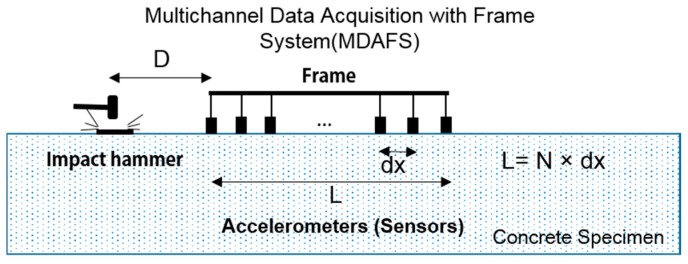
The scheme for an MASW test associated with MDAFS. D is near the offset; dx is the sensor spacing; and L is the offset range.

The scheme of MDAFS is presented in Figure 4. This makes it possible for a multichannel record to be obtained with a single test. All of the recorded signals are compiled to make an equivalent multichannel record for dispersion analysis. With this system, the signal from each impact is automatically streamed to the hard drive of a portable computer. The resulting multichannel record at different offsets, *u*(*x*,*t*), is automatically and objectively transformed to the frequency phase velocity domain with the MASW processing technique; the plane wave transformation is in the following equation [20,21].

(6) $$S(\omega ,c_{P})={\int e}^{-i(\omega /c_{P})x}U(x,\omega )dx$$

where *U*(*x*,*ω*) is the normalized complex spectrum obtained from the Fourier transformation of *u*(*x*,*t*), *ω* is the angular frequency and *C_P_* is the phase velocity.

*S*(*ω*,*C_P_*) is the stack amplitude for each *ω* and *C_P_*, which can be viewed as the coherency in the linear arrival pattern along the offset range for that specific combination of *ω* and *C_P_*. Thus, it is possible to extract multimodal dispersion curves from a multichannel dataset. All coherent phase velocity patterns are mapped with respect to their relative energy level. Therefore, the phase velocity image shows how the total seismic energy is distributed between different frequencies and phase velocities.

As a next step, the measured Lamb wave dispersion curves are matched to theoretical dispersion curves. In the general inversion analysis of MASW for the characterization of soil profile and asphalt pavement, by changing the density, elasticity, thickness and Poisson’s ratio, the theoretical dispersion curves are matched (either manually or automatically) to the experimental phase velocity image [20,21]. However, this study utilizes the measured Poisson’s ratio by the standard test or the representative value (=0.2) for the normal-strength concrete, because the change of the dispersion curve is negligible in the general range of Poisson’s ratio 0.15~0.25 for the concrete [17]. Moreover, Popovics [22] showed that both the static and dynamic Poisson’s ratio vary across a broad range of values for concrete, usually between 0.15 and 0.25, and that the dynamic and static values of Poisson’s ratio are not strongly related to each other.

Lastly, the elasticity can be estimated by Poisson’s ratio and the Rayleigh wave velocity extracted from the A0 Lamb mode in the high frequency range; then, the thickness of the target structure can be estimated by Equation (5), combing the estimation of P-wave velocity from R-wave velocity and the S1-ZGV Lamb mode frequency. This whole procedure can be done in the field in just a few minutes, provided that normalized dispersion curves have been calculated in advance (as described in the previous section). Thus, the Rayleigh wave velocity and thickness can be estimated from the A0 and S1 modes in the dispersion curve. The complete analysis scheme is discussed below.

## 4. Experiments

### 4.1. Test Specimens

**Figure 5:**
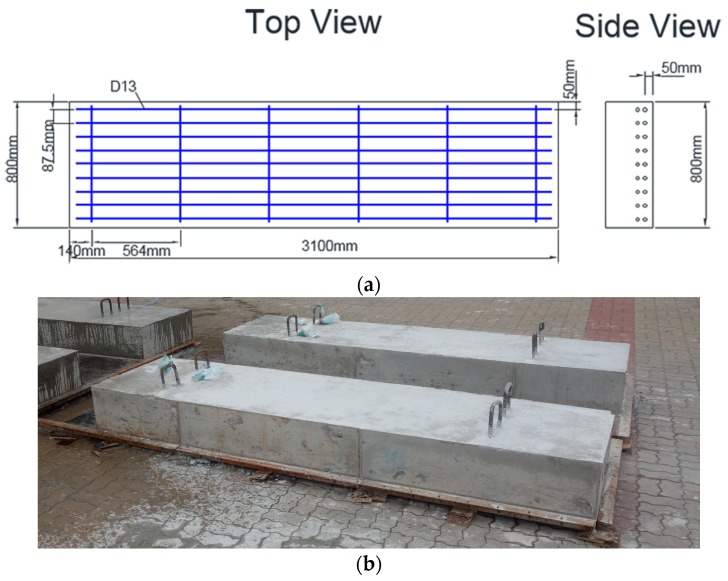
Concrete wide beam test specimen. (**a**) The planar and side views; (**b**) the test specimen after casting.

To investigate the practical effectiveness of our MASW scheme for assessing the conditions of concrete structures, we carried out a series of MASW tests on eight concrete wide beams (W1, W2, …, W8) cast from the same batch of concrete, as shown in Figure 5. All of the specimens are 800 mm × 3100 mm with a nominal thickness of 300 mm. They all contain transverse and longitudinal 13 mm-diameter reinforcements with 564 mm and 87.5 mm spacings, respectively, at a depth of 250 mm.

The specified w/c of the concrete mixture was 0.70 with an entrained air content of 6%. The mixture contained Type I cement (Ssangyong Cement Co., Ltd., Yeongwol, Korea) and limestone aggregates with a maximum coarse aggregate size of 25 mm. At the time of concrete placement, the slump was measured to be 150 mm, and the density was 2250 kg/m^3^. The specimens were moist-cured for seven days by covering them with saturated burlap with a bleed hose and plastic sheeting. Beyond the eighth day, the specimens were kept in laboratory air.

We determined the mechanical properties of the concrete by conducting tests performed on ten 150 mm × 300 mm standard cylinders (Kumhohoning, Seoul, Korea) prepared from the same batch of concrete. For the same conditions as the W1, W2, …, W8 concrete wide beams, we air-cured the standard specimens for 28 days. We determined the compressive strength using ASTM C-39 (ASTM International, West Conshohocken, PA, USA) [23] and found the modulus of elasticity (Ec) and Poisson’s ratio (v) with ASTM C-469 (ASTM International, West Conshohocken, PA, USA) [24]. The average results for the 28-day tests yielded 14.84 MPa for the strength, 17.68 GPa for the modulus of elasticity and 0.167 for static Poisson’s ratio. Furthermore, the dynamic modulus (Ed) was 21.30 GPa, as determined by following the empirical relationship proposed by Lydon and Balendran [25].

*E_c_* = 0.83 *E_d_* (GPa) (7)

### 4.2. Test Methods

The purpose of this study is to verify the practical effectiveness of our proposed MASW in the easy and simple manner associated with the MDAFS. First, the condition assessment and dimension identification of concrete structures were carried out by MASW. Second, the P- and S-wave velocities were also measured to verify the test results.

#### 4.2.1. MASW Test for R-Wave Velocity and Thickness Measurement

**Figure 6:**
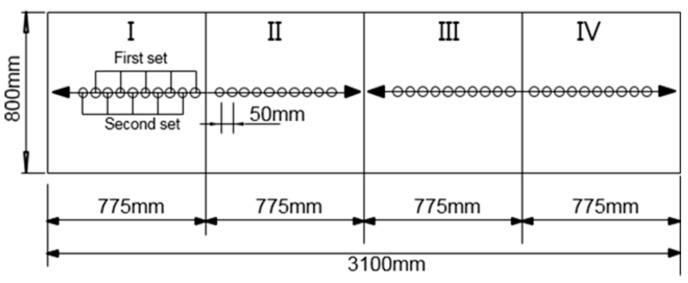
A planar view of the four test paths for the MASW test. The O marks indicate the sensor positions in each path, and the arrows show the direction of each test.

We applied an MDAFS testing configuration to consistently identify the R-wave velocity and thickness of eight test specimens along four different paths (I, II, III and IV), as shown in Figure 6.

**Figure 7:**
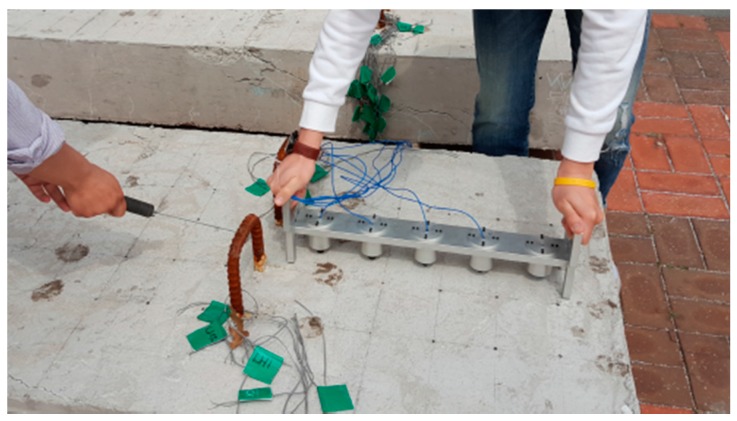
The MDAFS testing configuration for the MASW along predefined paths on the surface of a concrete wide beam.

We used a ball-type impact hammer, which is capable of measuring the force historical data for each MASW test. The forcing function associated with the impact exhibits consistent and broad spectral content, ranging from Direct Current (DC) to 30 kHz. We used an accelerometer (PCB 353B16) (PCB Piezotronics, Inc., Depew, NY, USA) with a ±5% frequency range of 1 kHz to 60 kHz and a resonance frequency around 70 kHz. Due to the number of sensors, five accelerometers with 10 cm spacing were equipped with the MDAFS. A dry point coupling between the accelerometer and concrete surface was done by both the thin rubber tip on each sensor and spring loading from the frame. For improved application of MASW, the MDAFS was carried out twice with a 5 cm offset along the same path. In this manner, 10 sensor measurements with 5 cm spacing along the test line could be completed. Figure 7 shows the MDAFS testing configuration. As a next step, we stabilized the signals using a signal conditioner (PCB 482C16) (PCB Piezotronics, Inc., Depew, NY, USA) and digitized them at a sampling frequency of 1 MHz using an NI-PXI 5105 oscilloscope (National Instruments Co., Austin, TX, USA). The basic concept in our MASW setup is similar to the classical procedure, which consists of one receiver (geophone) and the multiple hammer impacts in the geotechnical engineering [20,21]. However, the frequency range of the sensor, impactor and Data Acquisition (DAQ) in our test is tailored to the concrete structures with normal or high strength. Furthermore, the simultaneous data record by the multiple sensors associated with the high sampling rate makes it possible to have a clear experimental dispersion curve in the high frequency range.

The multichannel record consists of 10 time series (called traces) from the receivers in an ordered manner. The overall MASW data processing and analysis consists of four steps: (1) the multichannel data record; (2) construction of the dispersion image and extraction of the signal dispersion curve; (3) identification of several fundamental Lamb wave modes; and (4) estimation of the R-wave velocity and impact-echo frequency from A0 and S1 modes. All of these steps can be fully automated. Components of the necessary dispersion curve, such as the A0, A1, S0 and S1 modes, are then extracted from the accumulation pattern. The extracted dispersion curve is finally used as a reference to identify the R-wave velocity and thickness, corresponding to A0 and S1, respectively. For each test, we obtained time signals that were 10 ms in duration with a 1-MHz sampling rate. Because the amplitude of the input forcing function at each test point across the concrete surface is inherently inconsistent, we normalized the amplitude of each time signal with respect to the maximum peak of the input force from the force data measured by the impact hammer; this provides more consistent MASW data.

**Figure 8:**
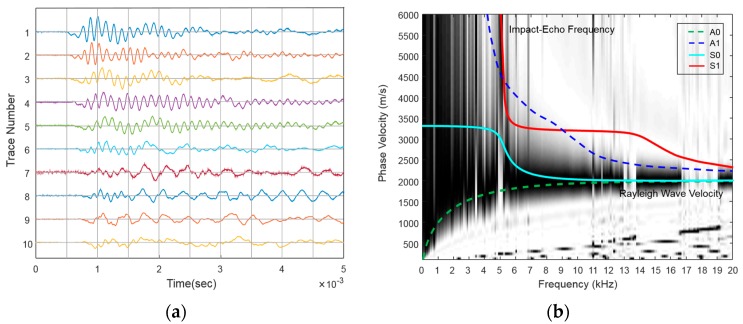
Experimental MASW results along Path III in specimen W2. The computed A0, A1, S0 and S1 Lamb modes corresponding to the specimen depth overlap on the dispersion curve image. (**a**) The signals in the time domain; (**b**) dispersion curve.

Figure 8 shows the experimental MASW results along Path 3 (III) in the W2 beam and exhibits dispersion images and Lamb wave multimodes up to 20 kHz. The Lamb wave curves in the single layer must be fit to one of the responses. The A0 and S0 modes converge beyond 10 kHz into a phase velocity of 1878 m/s, which corresponds to the R-wave. The P-wave velocity (3279 m/s) can be estimated from the R-wave velocity by using the elastic relationship described below Equation (8) (with Poisson’s ratio equal to 0.167, which was already obtained from ASTM C-469 (ASTM International, West Conshohocken, PA, USA). Furthermore, Poisson’s ratio can be verified by matching the experimental dispersion curves with the theoretical Lamb wave modes, which is done by changing the elastic properties (e.g., the elasticity, density and Poisson’s ratio). This process is typically referred to as inversion analysis.

(8) $$V_{P}=\frac{1+v}{0.87+1.12v}\sqrt{\frac{2(1-v)\;}{1-2v}}V_{R}$$

To estimate the thickness, the S1-ZGV Lamb mode frequency that corresponds to the impact-echo mode (thickness mode) is identified to be 5.1 kHz (Figure 8). Using the impact-echo Equation (5), the thickness is then calculated to be 0.309 m.

#### 4.2.2. P-Wave Velocity Measurement

**Figure 9:**
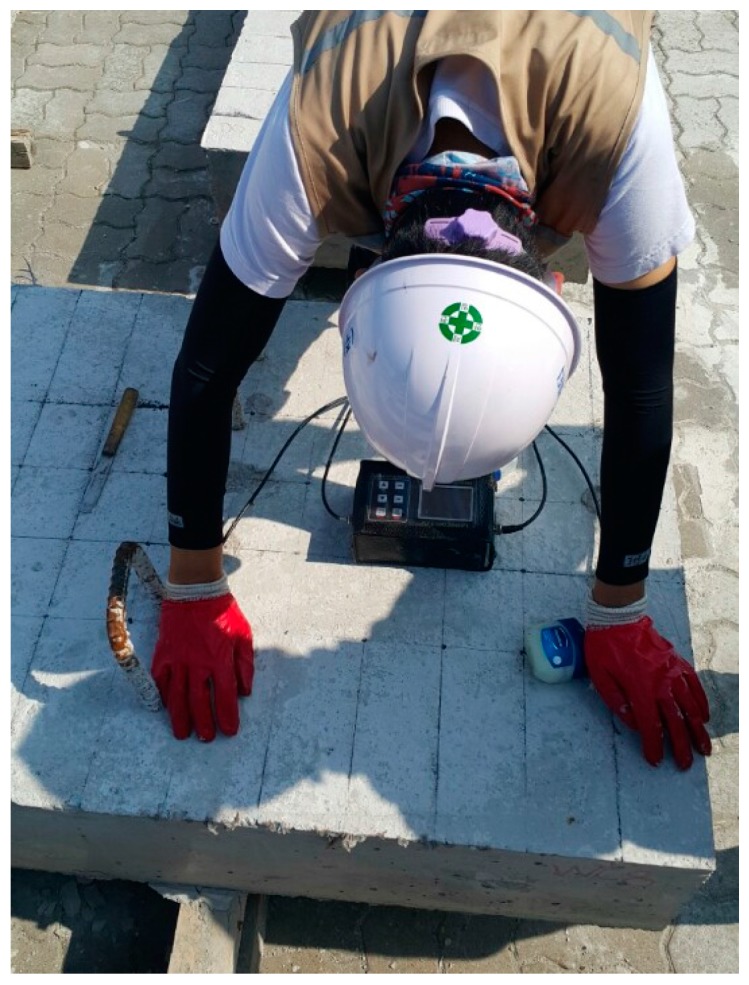
P-wave measurement in a grid on the surface of a concrete wide beam.

For reference, the P-wave velocity of concrete *V_P_* (according to British Standard (BS) 1881) [26] was measured using a pair of P-wave transducers (MK-954 transmitters and receivers) (MKC Co., Seoul, Korea) that were connected to a pulse-receiver (Ultracon-170) (MKC Co., Seoul, Korea). The transmitter was driven by a 200-V square pulse, generating a transverse ultrasonic pulse of 52 kHz. The receiver measured the transient stress waves through the surface of each concrete beam (Figure 9).

We performed P-wave measurements with a digital scope board (National International Co., Austin, TX, USA) synchronized with the pulser-receiver unit; therefore, data collection started at the time of pulse application. Measurement of the flight time was affected by electrical noise superimposed with the waveform. Therefore, to minimize random errors associated with identifying the arrival time, we performed wave-averaging on 128 waveforms. The first step in identifying the flight time from the waveform was to establish a baseline. Afterward, the waveform was further smoothed using a 10-point moving average filter.

**Figure 10:**
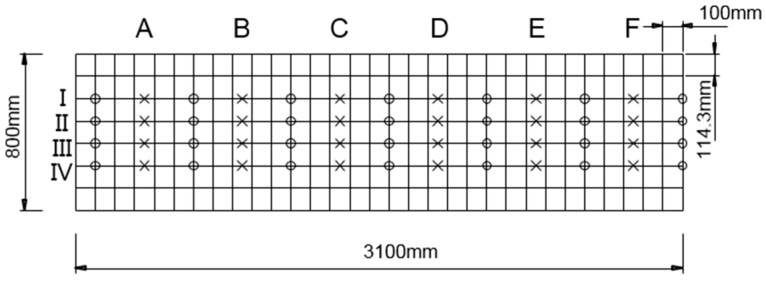
A planar view of the 114 × 100 mm^2^ test point grid for P-wave measurements. The test was carried out on the grid locations. The O and X marks indicate the positions of the transducers and average measurements, respectively. All dimensions in millimeters.

We made a total of 24 indirect measurements on each concrete wide beam using a coordinate system drawn on the beam’s surface (Figure 10). The coordinate system consisted of a primary grid with 100 × 113 mm^2^ spacing. The gridlines were labeled along the width of the specimens as Axes A, B, C, D, E and F and along the length of the specimens as Axes I, II, III and IV. We made indirect measurements longitudinally along the lettered axes. We placed the transmitter and receiver transducers at the grid nodes and measured the average P-wave velocity between them.

We acquired the transmitter and conditioned receiver signals using a high-speed (1-MHz sampling rate) analog-to-digital data acquisition board (National International Co., Austin, TX, USA). We developed a computer algorithm based on a fixed threshold level to determine the time of flight using the digitally-acquired waveforms.

## 5. Results and Discussion

### 5.1. Experimental Results from the MASW Tests

Generally, a variety of experimental data along each test line from the MASW test is presented in the dispersion curves of various Lamb wave modes. Then, the data are fit to analytically-computed fundamental A0, A1, S0 and S2 Lamb modes with an iterative procedure by changing the Poisson’s ratio, density and elasticity of the material. However, in this study, a much simpler procedure than the general approach was applied. This consists of four steps: (1) identification of the Rayleigh wave velocity, which is the asymptotic value of the phase velocity of the A0 mode at increasing frequencies in the dispersion curve; (2) estimation of the P-wave velocity with the elastic wave relationship associated with the Poisson’s ratio by Equation (8); (3) identification of the S1-ZGV Lamb mode frequency; and (4) estimation of the thickness with the impact-echo Equation (5). Although the measurement of Poisson’s ratio is not carried out, the average value in the range of 0.15 and 0.25 for the normal strength concrete can be used because the dispersion curves are insensitive to changes in Poisson’s ratio [17]. In addition, the ZGV frequency of the S1 mode can be reconfirmed by relating the maxima associated with the fundamental thickness resonance in the summed frequency domain amplitude spectra. Thus, the proposed analysis technique is straightforward and can be easily applied.

**Figure 11:**
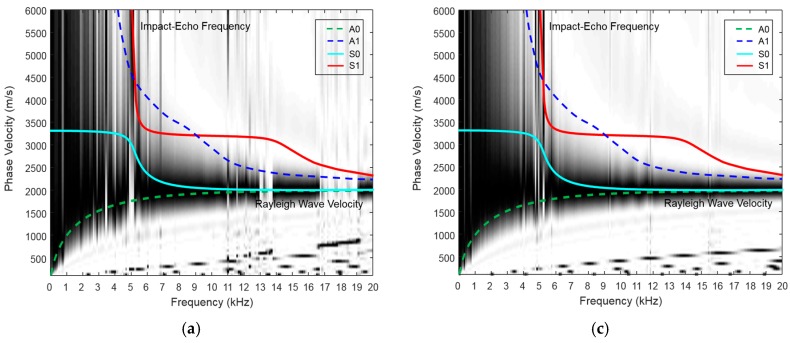

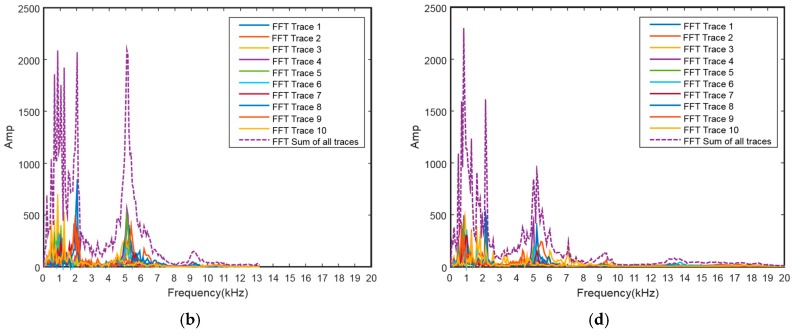
Experimental MASW results for two concrete wide beams (W2 and W4). A Poisson’s ratio of 0.16 was measured for the concrete cylinder. (**a**) Phase velocity dispersion curve along path III in the W2 beam; (**b**) summed amplitude spectra along path III in the W2 beam; (**c**) phase velocity dispersion curve along path II in the W4 beam: (**d**) summed amplitude spectra along path II in the W4 beam.

Figure 11 shows the test results from two representative samples obtained by the proposed procedure. This figure easily confirms that the asymptotic phase velocity over 10 kHz approaches the Rayleigh wave velocity (near 1900 m/s) and that the impact-echo frequency (near 5 kHz) in the frequency domain amplitude spectra is well-matched with the ZGV frequency of the S1 mode in the dispersion curve. In addition, the cut-off region (white area between 5 and 6 kHz) in the A0 mode also guarantees the existence of the ZGV frequency in the S1 mode. The amplitude at the impact-echo frequency in the frequency domain amplitude spectra along Path III in the W2 beam indicates one representative thickness, whereas the amplitude along Path II in the W4 beam is considerably lower; this is the case because the thickness cannot be consistent along Path III. Utilizing the impact-echo Equation (5) with the impact-echo frequency and the P-wave velocity estimated from the R-wave velocity, the thickness is estimated to be about 0.3 m; this is close to the actual thickness.

**Figure 12:**
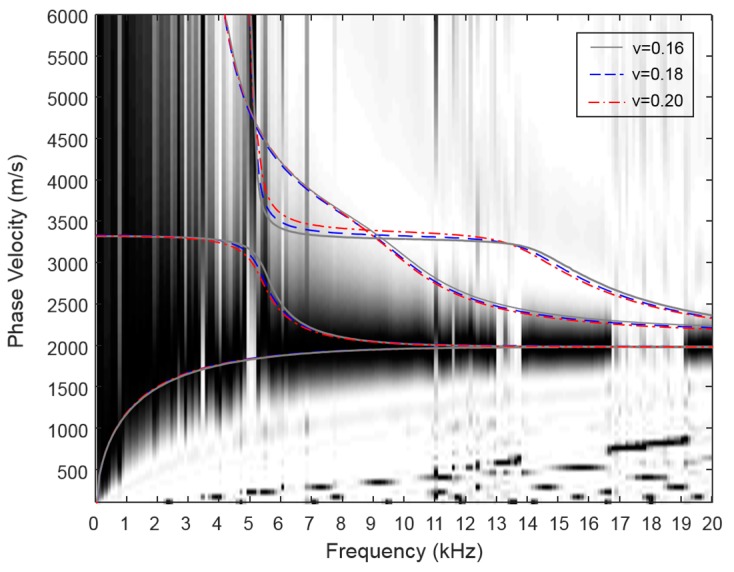
The theoretical dispersion curves on the experimental dispersion spectrum for three values of Poisson’s ratios in the W2 concrete wide beam.

Figure 12 shows the change of the theoretical dispersion curve for the three values of Poisson’s ratio (0.16, 0.18 and 0.20) on the experimental dispersion spectrum along Path III in the W2 beam. The phase velocities (Rayleigh wave velocity) of the A0 Lamb mode at the 20 kHz for the Poisson’s ratios 0.16 and 0.20 are 1878 m/s and 1860 m/s, and S1-ZGV Lamb wave frequencies for all of the Poisson’s ratios are converge to 5100 kHz. Thus, the dispersion curve is not sensitive to a general range of Poisson’s ratio for the concrete.

The data from the dispersion curve and the summed amplitude spectra below 3 kHz show lots of noise and several large peaks that are not related to our interests (*i.e.*, the Rayleigh wave velocity and ZGV frequency of the S1 mode). These could be caused by the bending modes of the beam itself (flexural behavior) and/or by interference between the Rayleigh wave and the P-wave close to impact, which is typically considered to be a near-field effect. Fortunately, separating this noise from the impact-echo frequency (5 kHz) was unnecessary in our specimens. However, for the structures for which the width to depth ratio is small (*i.e.*, far from the plate-like structure), signal processing (e.g., filtering) can be necessary. Thus, the MASW test has certain limitations in applying to no plate-like structures. Table 1 shows a summary of the MASW results for all of the specimens (W1 to W8). Due to the advantage of multiple sensors, the measured Rayleigh wave velocities and thicknesses are consistent.

### 5.2. Experimental Data of P-Wave and the Statistical Reliability

To verify the MASW results, the P-wave velocities were also measured. The data reliability in the measurement of the P-wave velocity were assured by calculating the averaged flying time based on 128 waveforms. The range of the P-wave velocities for all of the specimens is 3263 m/s to 3471 m/s. This range is comparable to the typical one of sound concrete [27]. Furthermore, we investigated the statistical reliability analysis for all of the Ultrasonic Pulse Velocity (UPV) data, including the R-wave velocities (by MASW) and the P-wave velocities. This was done in order to evaluate variations in the experimental data. We used the sample size, mean and standard deviation of the UPV for the basic inputs, as summarized in Table 2.

The statistical data show that the standard deviation and COV decrease in the order of R- and P-waves, which means that the UPV data are more statistically stable in the order. According to American Concrete Institute (ACI) 214R, the COVs of the R-wave velocities are excellent (<3.0), and the COVs of the P-wave velocities are fair (5.0 to 6.0) [28]. In summary, based on the respective means and standard deviations, we infer that all of the UPV data are statistically reliable.

### 5.3. Comparison with the P-Wave Velocities and the Actual Depth

We checked the accuracy of our experimental MASW data by comparing the R-wave velocity and thickness with the measured P-wave velocities (obtained from traditional UPV measurements) and the actual thickness. Table 3 shows the comparison between P-wave velocities (estimated from the R-wave velocity) and the measured P-wave ones. It also compares the estimates from the MASW data to the actual thickness. The average error of the comparisons with the measured P-wave velocities is 2.09%. Furthermore, the error with regard to the thickness estimate is only 2.17%. Thus, the results from the MASW test are comparable to the results of other UPV tests. The practical application of MASW that is proposed in this study can be an effective method to identify the dimensions and elastic wave velocities.

In an elastic solid medium, the velocities of P- and R-wave propagation depend on elastic constants and the mass density of the material. The relations among P- and R-wave velocities can be expressed by Equations (9) and (10) [3]. From these relationships, the elastic properties, such as the elasticity and strength, can be estimated. We checked the accuracy of our experimental data by comparing the dynamic moduli from R- and P-wave velocities by the MASW and the ultrasonic test to one determined from the static modulus in Table 4. It shows that errors of the dynamic moduli for R- and P-wave velocities are 4.37% and 8.82%, respectively, and that estimation of the dynamic elasticity by the proposed MASW test is quite accurate.

(9) $$V_{P}=\sqrt{\frac{E(1-v)}{\rho (1+\nu )(1-2v)}}$$

(10) $$V_{R}=\frac{0.87+1.12v}{1+\upsilon }\sqrt{\frac{E}{\rho }\frac{1}{2(1+\nu )}}$$

where *V_P_* and *V_R_* are the velocities of P- and R-waves, respectively, and *E*, *ρ* and *ν* are dynamic elasticity, density and Poisson’s ratio, respectively.

## 6. Conclusions

The practical application of MASW associated with the MDAFS testing configuration allowed us to create reliable dispersion curves of several fundamental Lamb wave modes. Furthermore, the summed amplitude in the frequency domain equivalent to the IE test results could be extracted from the MASW data. The unique features that make the MASW approach particularly efficient are related to its simple and objective Lamb wave analysis, as well as to its effective data acquisition system. All of the results by the simple Lamb wave analysis associated with the practical MASW test agrees well with data from the traditional UPV test and with the actual thickness values. Thus, this analysis has been verified to be a robust and promising test method for identifying the elastic wave velocities and dimensions. In addition, all of our experimental data are statistically stable and significant. Based on the data presented in this study, we draw the following conclusions.

- The practical MASW approach associated with the multiple sensors and instrumented impact hammer with the appropriate frequency range in characterizing the elastic properties of concrete enables to complete the effective dispersion spectrum especially for the A0 and S1 lamb modes.
- The proposed Lamb wave analysis provides a simpler procedure than the inversion analysis in the general MASW, which has a complicated iterative process that requires changing various material properties such as the density, elasticity, thickness, and Poisson’s ratio. Moreover, it showed good accuracy with an error less than 3% when compared to the UPV test results and the actual dimensions.
- The experimental data from MASW are more statistically stable than data obtained from other UPV tests because of improvements in the measurement equipment and analysis; this improvement is associated with the fact that multiple sensors are used.
- We verified that the MASW data are reliable and can be used for the identification of the dimensions, as well as for the estimation of elastic wave velocities for the condition assessment. Our experimental results offer practical guidelines for the application of the MASW method to concrete structures.

## Figures and Tables

**Table 1:** The experimental results of MASW along four paths in all of the specimens. VR, Raleigh wave velocity.

**Specimen**	**W1**				**W2**				**W3**				**W4**			
Test point	I	II	III	IV	I	II	III	IV	I	II	III	IV	I	II	III	IV
VR (m/s)	1842	1842	1829	1899	1828	1873	1878	1810	1840	1892	1881	1842	1859	1874	1833	1862
Avg.* VR	1853				1847				1864				1857			
Vp** (m/s)	3216	3216	3194	3316	3192	3271	3279	3161	3213	3304	3284	3216	3246	3272	3201	3251
f_IE_ (Hz)	5100	5100	5300	5600	5700	5300	5100	5500	5500	5100	5400	5000	5000	5600	5100	5500
Depth (cm)	0.303	0.303	0.289	0.284	0.269	0.296	0.309	0.279	0.280	0.311	0.292	0.309	0.312	0.280	0.301	0.284
Avg.* thickness	0.295				0.287				0.298				0.294			
**Specimen**	**W5**				**W6**				**W7**				**W8**			
Test point	I	II	III	IV	I	II	III	IV	I	II	III	IV	I	II	III	IV
VR (m/s)	1866	1905	1845	1880	1884	1858	1874	1879	1869	1888	1880	1832	1885	1862	1856	1885
Avg.* VR	1874				1874				1867				1872			
Vp** (m/s)	3258	3326	3222	3283	3290	3244	3272	3281	3264	3297	3283	3199	3291	3251	3241	3291
f_IE_ (Hz)	5500	5600	5400	5000	5100	5000	5100	5500	5000	5200	5600	5700	5500	5600	5400	5000
Depth (cm)	0.284	0.285	0.286	0.315	0.310	0.311	0.308	0.286	0.313	0.304	0.281	0.269	0.287	0.279	0.288	0.316
Avg.* thickness	0.293				0.304				0.292				0.292			

Avg.* = Average, Vp** = P-wave velocity estimated by Equation (8).

**Table 2:** Results of the statistical analysis for R-wave velocities.

Specimen		W1	W2	W3	W4	W5	W6	W7	W8	Total Avg.
R-wave by MASW	Sample size	4	4	4	4	4	4	4	4	4
	Avg. (*μ*) * (m/s)	1853	1847	1864	1858	1874	1874	1867	1872	1864
	Std (*σ*) ** (m/s)	31	34	27	19	25	11	25	15	23
	COV	1.7%	1.8%	1.4%	1.0%	1.3%	0.6%	1.3%	0.8%	1.3%
P-wave by UPV	Sample size	24	24	24	24	24	24	24	24	24
	Avg. (*μ*) * (m/s)	3471	3359	3275	3327	3272	3263	3274	3341	3323
	Std (*σ*) ** (m/s)	232	231	207	302	157	153	176	122	197
	COV ***	6.7%	6.9%	6.3%	9.1%	4.8%	4.7%	5.4%	3.7%	5.9%

Avg. (*μ*) * = average velocity; Std (*σ*) ** = standard deviation of velocity; COV *** = coefficient of the variable (=*σ*/*μ* × 100 (%)).

**Table 3:** Comparison of MASW results with the measured P-wave velocities and the actual thickness.

Specimen	MASW			Vp (m/s)	Actual Thickness (m)	Error (%)	
	VR (m/s)	Vp * (m/s)	Thickness (m)			P-Wave	Thickness
W1	1853	3236	0.295	3471	0.3	6.77%	1.76%
W2	1847	3226	0.287	3359	0.3	3.98%	4.21%
W3	1864	3254	0.298	3275	0.3	0.62%	0.66%
W4	1858	3244	0.294	3327	0.3	2.50%	1.86%
W5	1874	3272	0.293	3272	0.3	0.01%	2.42%
W6	1874	3272	0.304	3263	0.3	0.27%	1.28%
W7	1867	3260	0.292	3274	0.3	0.41%	2.64%
W8	1872	3269	0.292	3341	0.3	2.17%	2.50%
Average	1864	3254	0.294	3323	0.3	2.09%	2.17%

Vp * = P-wave velocity estimated by Equation (8).

**Table 4:** Comparison of dynamic moduli from dynamic and static tests.

Measurement	Dynamic Test		Static Test				Error (%) of Ed
	Avg. (m/s)	Ed * (GPa)	v	Fck (Mpa)	Ec (GPa)	Ed (GPa)	
R-wave by MASW	1864	22.23	0.16	14.84	17.68	21.3	4.37%
P-wave by UPV	3323	23.18	0.16	14.84	17.68	21.3	8.82%

Ed * = dynamic modulus estimated by Equations (9) and (10).
